# Blockade of Pachytene piRNA Biogenesis Reveals a Novel Requirement for Maintaining Post-Meiotic Germline Genome Integrity

**DOI:** 10.1371/journal.pgen.1003038

**Published:** 2012-11-15

**Authors:** Ke Zheng, P. Jeremy Wang

**Affiliations:** Department of Animal Biology, Center for Animal Transgenesis and Germ Cell Research, University of Pennsylvania School of Veterinary Medicine, Philadelphia, Pennsylvania, United States of America; Cornell University, United States of America

## Abstract

Piwi-interacting RNAs are a diverse class of small non-coding RNAs implicated in the silencing of transposable elements and the safeguarding of genome integrity. In mammals, male germ cells express two genetically and developmentally distinct populations of piRNAs at the pre-pachytene and pachytene stages of meiosis, respectively. Pre-pachytene piRNAs are mostly derived from retrotransposons and required for their silencing. In contrast, pachytene piRNAs originate from ∼3,000 genomic clusters, and their biogenesis and function remain enigmatic. Here, we report that conditional inactivation of the putative RNA helicase MOV10L1 in mouse spermatocytes produces a specific loss of pachytene piRNAs, significant accumulation of pachytene piRNA precursor transcripts, and unusual polar conglomeration of Piwi proteins with mitochondria. Pachytene piRNA–deficient spermatocytes progress through meiosis without derepression of LINE1 retrotransposons, but become arrested at the post-meiotic round spermatid stage with massive DNA damage. Our results demonstrate that MOV10L1 acts upstream of Piwi proteins in the primary processing of pachytene piRNAs and suggest that, distinct from pre-pachytene piRNAs, pachytene piRNAs fulfill a unique function in maintaining post-meiotic genome integrity.

## Author Summary

Small non-coding RNAs play critical roles during development and in disease. The integrity of the germline genome is of paramount importance to the wellbeing of offspring and the survival of species. Piwi-interacting RNAs (piRNAs) are a class of small non-coding RNAs abundantly expressed in the gonad. Compared to microRNAs and small-interfering RNAs (siRNAs), the biogenesis and function of piRNAs remain poorly understood. Here we have identified MOV10L1, a putative RNA helicase, as a master regulator of piRNA biogenesis in mouse. We find that production of pachytene piRNAs requires MOV10L1. Blockade of pachytene piRNAs disrupts germ cell development and results in defects in post-meiotic genome integrity. Therefore, mutations in MOV10L1 and other piRNA pathway components may contribute to male infertility in humans.

## Introduction

Piwi-interacting RNAs (piRNAs) are a diverse class of gonad-specific small interfering RNAs that bind to members of the Piwi subfamily of Argonaute proteins. One common function of piRNAs in all species studied so far is the silencing of transposable elements, which is essential for the protection of genome integrity during germ cell development [1]–[3]. Distinct from miRNAs and siRNAs in origin, length, structure, and biogenesis, piRNAs are generated by dicer-independent processing of long precursor transcripts, however, the precise mechanisms of their biogenesis remain largely unclear [4], [5]. In mice, the Piwi family has three members: *Miwi* (*Piwil1*), *Mili* (*Piwil2*), and *Miwi2* (*Piwil4*). These Piwi genes exhibit different developmental expression patterns in testis. While *Miwi2* is expressed in fetal and perinatal germ cells [6], the expression of *Miwi* is restricted to pachytene spermatocytes and round spermatids in adult testes [7]. *Mili* is expressed from the fetal germ cell stage onwards through the round spermatid stage [8]. Two developmentally distinct populations of piRNAs are expressed in mouse male germ cells at the pre-pachytene and pachytene stages. Pre-pachytene piRNAs are mostly derived from transposable elements and are associated with MILI and MIWI2 in fetal and perinatal male germ cells [6], [9], [10]. Pachytene piRNAs originate from ∼3000 genomic clusters [11] and bind to both MILI and MIWI [12]–[17]. Interestingly, more than 90% of MILI- and MIWI-bound pachytene piRNAs shared identical 5′end sequences [18]. As a result, most MILI- and MIWI-bound pachytene piRNAs map to the same genomic clusters [18].

The biogenesis of piRNAs involves primary and secondary processing mechanisms [1], [2]. Pre-pachytene piRNAs derive from precursor transcripts that are cleaved into putative primary piRNA intermediate molecules by a yet unknown primary processing mechanism, followed by loading onto MILI for further processing. In embryonic germ cells, the endonuclease (slicer) activity of MILI is required for the secondary piRNA processing mechanism, which amplifies MILI-bound piRNAs through an intra-MILI ping-pong loop and generates all MIWI2-bound secondary piRNAs [19]. In this feed-forward ping-pong model, Piwi proteins with piRNAs complimentary to retroelement-derived transcripts drive transcript cleavage and piRNA amplification [6], [9], [10], [19]. In contrast, the biogenesis of pachytene piRNAs only engages the primary processing mechanism, i.e. the presumptive cleavage by an unknown nuclease and eventual processing of the precursor transcript into mature piRNAs [5], [17], [20], [21]. Therefore, pachytene piRNAs provide a simple and ideal system for dissecting the mysterious primary processing mechanism in mammals [11], [13]–[16].

We and others previously demonstrated that MOV10L1, a putative RNA helicase, interacts with all mouse Piwi proteins and is required for biogenesis of pre-pachytene piRNAs [22], [23]. MOV10L1 homologues are evolutionarily conserved among insects (Armi in *Drosophila melanogaster*), plants (SDE3 in *Arabidopsis thaliana*), and vertebrates (MOV10 and MOV10L1). *Arabidopsis* SDE3 is required for post-transcriptional gene silencing [24]. *Drosophila* Armi is essential for the maturation of RISC (RNA-induced silencing complex) and miRNA-mediated silencing [25], [26]. Armi is also relevant to the piRNA pathway, evident from the loss of specific piRNAs and the activation of retrotransposons in *armi* mutants [27], [28]. Specifically, Armi plays an essential role in the primary piRNA processing pathway [29]. In contrast to *Drosophila* and *Arabidopsis* with a single *Mov10l1* homologue, the vertebrate genome encodes two genes (*Mov10* and *Mov10l1*), which apparently arose by gene duplication. MOV10 is ubiquitously expressed and associates with Ago proteins, forming part of the purified human RISC [30], [31]. Depletion of MOV10 in cultured cells leads to reduced miRNA-mediated silencing [30]. We initially identified MOV10L1 as a putative RNA helicase that is specifically expressed in mouse germ cells [32], [33]. Disruption of *Mov10l1* leads to meiotic arrest, de-repression of transposable elements, and depletion of both MILI- and MIWI2-associated perinatal piRNAs [22], [23]. Apparently, MOV10 and MOV10L1 function in the miRNA and the piRNA pathway, respectively, due to specialization after gene duplication during vertebrate evolution.

The existing piRNA pathway mouse mutants either fail to deplete all pachytene piRNAs or exhibit meiotic arrest prior to the pachytene stage, leaving the biogenesis and role of pachytene piRNAs largely unexplored. Inactivation of either *Mili* or *Miwi2* causes postnatal meiotic arrest at the leptotene/zygotene stage in the male germline [8], [34]. Similarly, other piRNA pathway mutants, such as *Ddx4* (Vasa), *Mael*, *Gasz*, *Tdrd9*, *Mov10l1*, and *Mitopld*, also exhibit early meiotic arrest in males [22], [35]–[40]. Inactivation of *Miwi* leads to spermiogenic arrest at the round spermatid stage [7]. However, MILI-associated pachytene piRNAs are abundant in *Miwi*-deficient testes [17], [18]. Therefore, a mouse mutant containing pachytene spermatocytes, but lacking all pachytene piRNAs (both MILI- and MIWI-bound piRNAs) has not been available to specifically study the function of pachytene piRNAs. In this study, we have specifically and completely depleted the pachytene piRNA population in the male germline of *Mov10l1* mutant mice, uncovering a novel function for pachytene piRNAs in maintaining post-meiotic genome integrity.

## Results

### MOV10L1 Is Essential for Spermiogenesis

**Figure 1:**
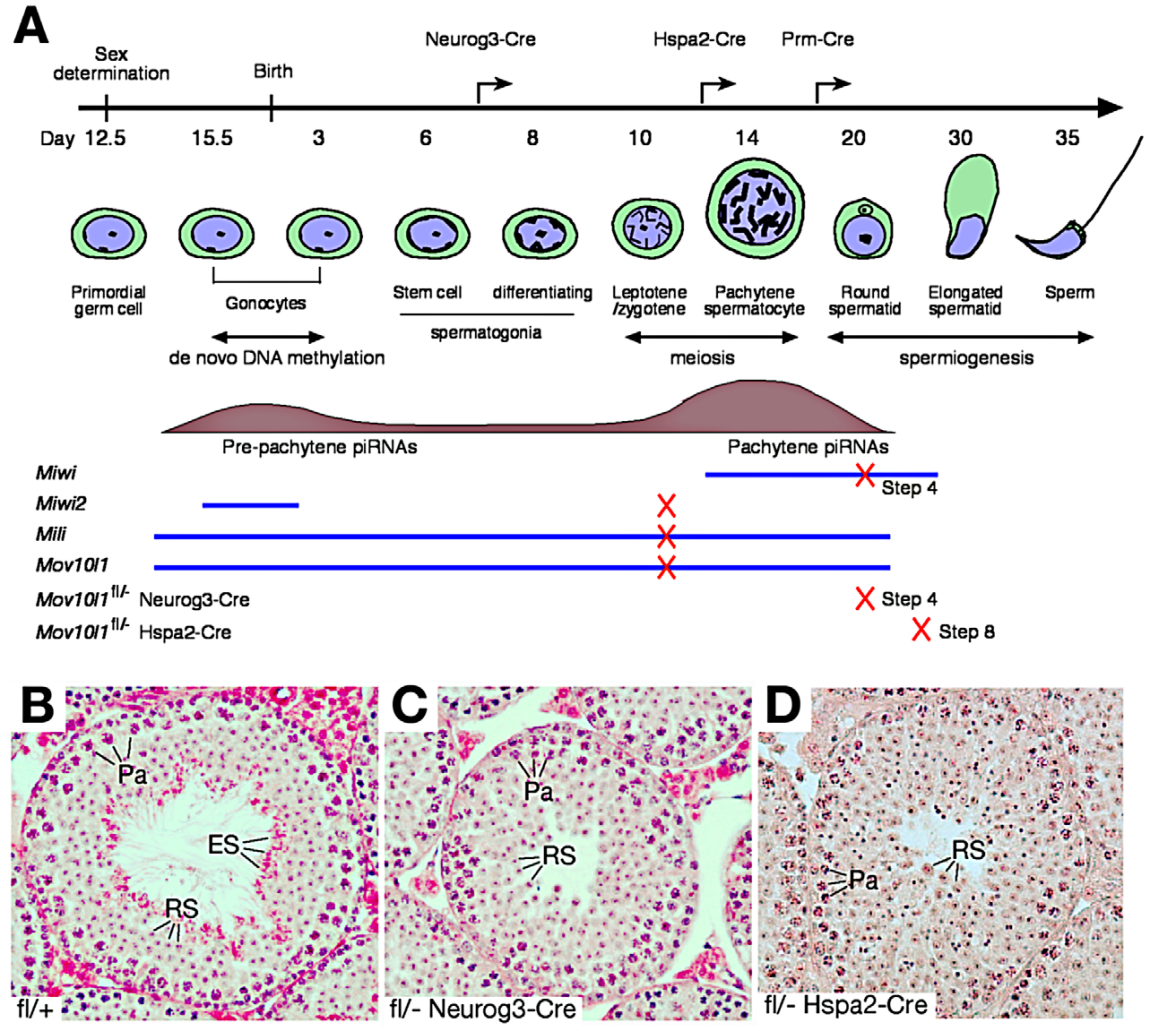
Post-natal disruption of *Mov10l1* leads to post-meiotic spermiogenic arrest. (A) Timeline of mouse spermatogenesis, with blue lines and tan histograms representing developmental expression patterns of three mouse Piwi proteins (MIWI, MIWI2, MILI) and MOV10L1, and pre-pachytene and pachytene piRNA populations, respectively. Crosses on lines mark the time point of spermatogenic arrest in the respective mouse mutant (4 ubiquitous null mutants and 2 *Mov10l1* conditional mutants). Arrows on the timeline bar indicate the onset of Cre expression in the different *Mov10l1* mutants generated. Disruption of *Mov10l1* by Prm-Cre did not cause spermiogenic arrest (Table S1 and Figure S3). (B–D) Histology of testes from adult wild-type (B), *Mov10l1* ^fl/-^ Neurog3-Cre (C), and *Mov10l1* ^fl/-^ Hspa2-Cre (D) mice. H&E staining was performed on testis sections as described in the Materials and Methods. Abbreviations: Pa, pachytene spermatocytes; RS, round spermatids; ES, elongated spermatids.

MOV10L1, a putative RNA helicase, interacts with all three mouse Piwi proteins, and is an essential component of the piRNA pathway [22]. To explore the biogenesis and function of pachytene piRNAs, we disrupted MOV10L1 function specifically during and after male meiosis using Cre-mediated inactivation of a conditional *Mov10l1* allele (*Mov10l1* ^fl^) (Figure 1A and Figure S1) at the following stages: after postnatal day 7 (Neurog3-Cre) [41], at the pachytene stage (Hspa2-Cre) [42], and in post-meiotic spermatids (Prm-Cre) [43].

Cre-mediated recombination of the conditional *Mov10l1* allele deletes the RNA helicase domain, producing a truncated protein termed MOV10L1Δ. In male *Mov10l1* ^fl/-^ Neurog3-Cre mice resulting from intercrosses of *Mov10l1* ^fl/fl^ mice with Neurog3-Cre mice [41], Cre-mediated disruption of *Mov10l1* was first detected in testes at postnatal day 9 (leptotene/zygotene spermatocytes), with a decrease in the abundance of the full-length MOV10L1 protein in the mutant testes compared with the wild type (Figure S2A). *Mov10l1* ^fl/-^ Neurog3-Cre males were sterile, with substantially smaller testes (140±10.5 mg/pair at 2–4 months of age) compared to age-matched wild-type mice (189±18.4 mg/pair) (Student's *t* test, p<0.0008). In contrast to seminiferous tubules from wild-type mice (Figure 1B), tubules from *Mov10l1* ^fl/-^ Neurog3-Cre mutant mice lacked elongated spermatids, while earlier germ cell stages including pachytene spermatocytes and round spermatids were present (Figure 1C). Acrosome staining with the anti-ACRV1 antibody identified spermiogenic arrest at the step 4 spermatid stage. Therefore, very different to the meiotic arrest observed in male germ cells with ubiquitous deletion of *Mov10l1* [22], [23], postnatal disruption of *Mov10l1* mediated by Neurog3-Cre causes post-meiotic spermiogenic arrest (Figure 1C), revealing that MOV10L1 plays an essential role during the post-meiotic stages of spermatogenesis.

To distinguish consequences of inactivation of MOV10L1 during the pachytene stage from those resulting from disruption at earlier stages such as in differentiating spermatogonia, we generated *Mov10l1* ^fl/-^ Hspa2-Cre mice in which Cre is expressed specifically in spermatocytes, particularly pachytene cells (Figure 1A) [42]. Deletion of MOV10L1 in *Mov10l1* ^fl/-^ Hspa2-Cre mice occurred by postnatal day 14, apparent from a decrease in the abundance of the full-length MOV10L1 protein in the mutant testes (Figure S2B). Notably, *Mov10l1* ^fl/-^ Hspa2-Cre males were also sterile. Although testes (159±24 mg/pair) from 2–3 month old *Mov10l1* ^fl/-^ Hspa2-Cre mice were slightly smaller than those from *Mov10l1* ^+/−^ males (182±26 mg/pair) (Student's *t* test, p<0.2), histological analysis revealed spermiogenic arrest at the round spermatid stage (Figure 1D). The most advanced spermatids in *Mov10l1* ^fl/-^ Hspa2-Cre males were late round spermatids at step 8. The arrest of spermiogenesis at early and late round spermatid stages in *Mov10l1* ^fl/-^ Neurog3-Cre and *Mov10l1* ^fl/-^ Hspa2-Cre mutant mice, respectively, demonstrates that MOV10L1 is required for the differentiation of post-meiotic germ cells. The temporal delay in the spermiogenic arrest in *Mov10l1* ^fl/-^ Hspa2-Cre testes is likely due to the late onset of Hspa2-Cre expression, which may allow residual MOV10L1 to persist longer.

The round spermatid arrest in *Mov10l1^fl^* ^/-^ Neurog3-Cre and *Mov10l1^fl^* ^/-^ Hspa2-Cre testes could be due to disruption of MOV10L1 function during the pachytene stage of meiosis, or at early spermatid stages. To define the requirement for MOV10L1 more precisely, we disrupted *Mov10l1* with Cre recombinase under the control of the protamine 1 (Prm) promoter, which is only expressed in post-meiotic spermatids [43]. *Mov10l1* ^fl/-^ Prm-Cre males exhibited normal fertility but a slight reduction in testis weight (Table S1). Histological analysis of testes from *Mov10l1* ^fl/-^ Prm-Cre males revealed normal spermiogenesis (Figure S3). These genetic studies demonstrate that disruption of MOV10L1 function at the pachytene stage causes spermiogenic arrest.

### Blockade of Pachytene piRNA Biogenesis in the *Mov10l1* Mutant

**Figure 2:**
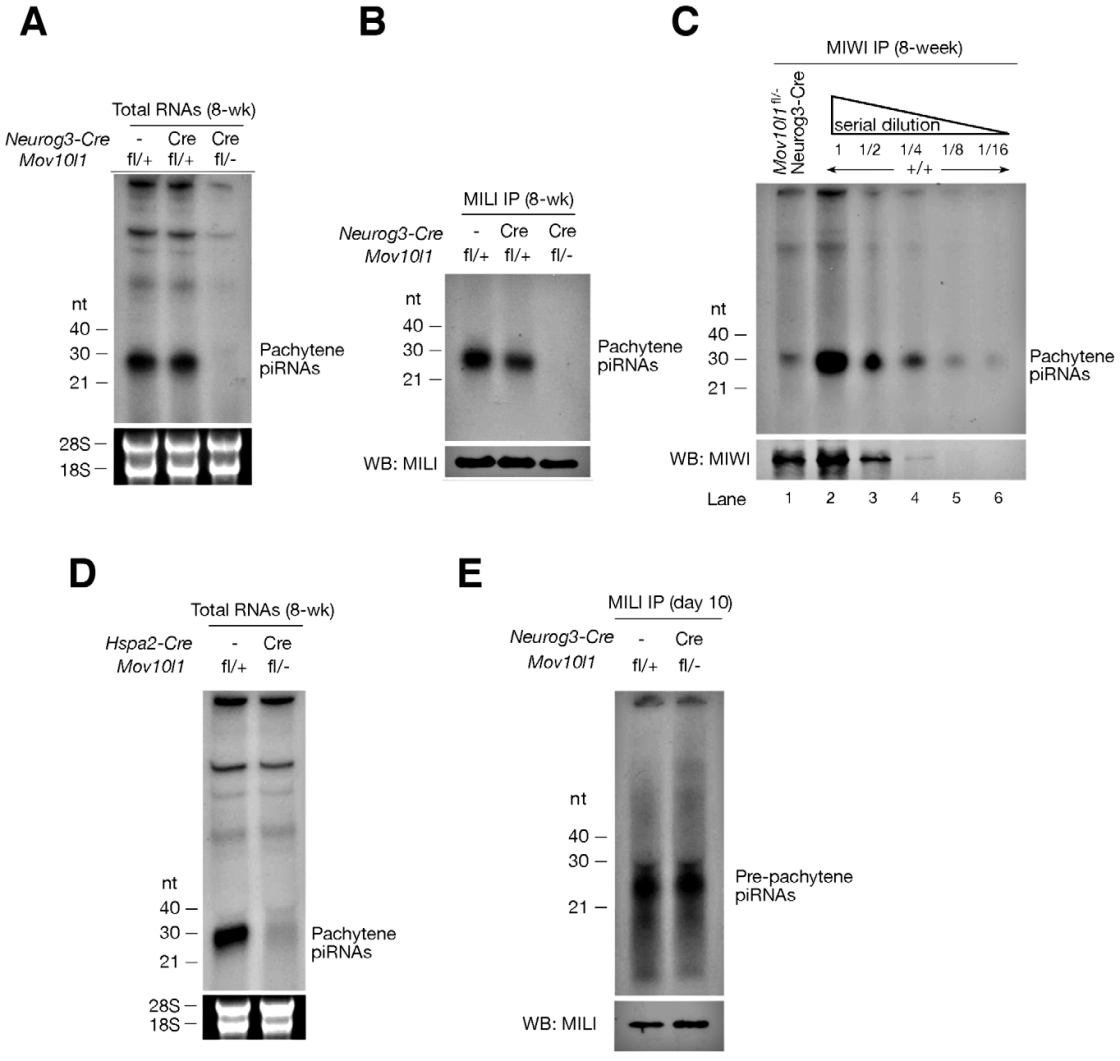
*Mov10l1* is required for biogenesis of pachytene piRNAs. (A) Depletion of pachytene piRNAs in mutant testes from *Mov10l1* ^fl/-^ Neurog3-Cre adult mice. Total RNAs were ^32^P-end-labelled and separated by denaturing polyacrylamide gel electrophoresis. 18S and 28S ribosomal RNAs served as a loading control. (B) MILI is devoid of pachytene piRNAs in *Mov10l1* ^fl/-^ Neurog3-Cre testes. MILI was immunoprecipitated from testicular extracts. One-tenth of the immunoprecipitated material was used for detection of associated piRNAs, and the remainder was used for Western blotting (WB) analysis of MILI. (C) MIWI immunoprecipitation on *Mov10l1* ^fl/-^ Neurog3-Cre and wild-type (+/+) testes, and serial dilutions (1∶2) of MIWI IP complexes. (D) Depletion of pachytene piRNAs in mutant testes from *Mov10l1* ^fl/-^ Hspa2-Cre adult mice. (E) MILI is loaded with pre-pachytene piRNAs in postnatal day 10 *Mov10l1* ^fl/-^ Neurog3-Cre testes.

**Figure 3:**
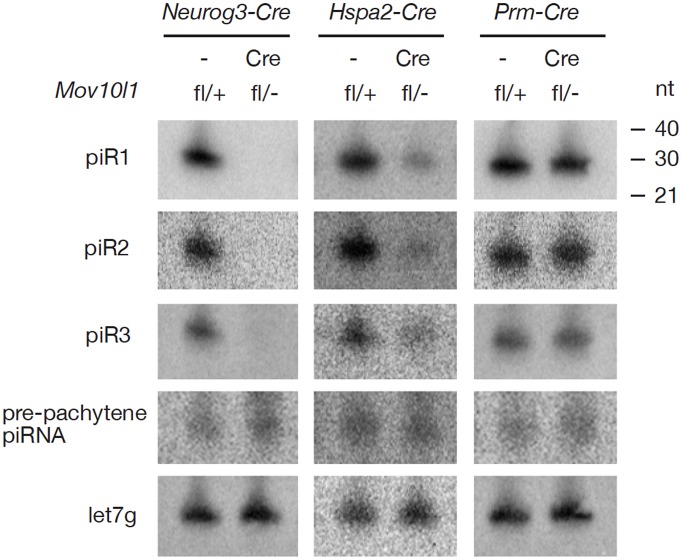
Northern blot analysis of individual pachytene piRNAs in conditional *Mov10l1* mutant testes. RNAs were prepared from adult testes and hybridized with radiolabeled probes specific for pachytene piR1, piR2, piR3, and a pre-pachytene piRNA. Hybridization with a let7g miRNA probe served as a loading control. The three individual pachytene piRNAs were absent in *Mov10l1* ^fl/-^ Neurog3-Cre testes, sharply reduced in abundance in *Mov10l1* ^fl/-^ Hspa2-Cre testes, and present in *Mov10l1* ^fl/-^ Prm-Cre testes, while the level of the pre-pachytene piRNA was not affected in either of these mutant testes.

Isolation and radiolabeling of total testicular small RNAs from adult *Mov10l1* ^fl/-^ Neurog3-Cre testes showed that mutant testes were devoid of pachytene piRNAs (Figure 2A). Immunoprecipitation experiments further revealed that both MILI- and MIWI-associated pachytene piRNAs were absent in the mutant (Figure 2B, 2C). As *Mov10l1* mutant testes contained less MIWI protein than wild-type testes, we performed serial dilutions of immunoprecipitated complexes to rule out the possibility that the observed loss of MIWI-bound piRNAs was due to the detection limit of the assay. However, MIWI-associated piRNAs were detectable in wild-type testes even when MIWI protein was not detectable (Figure 2C, lane 4), indicating a specific depletion of pachytene piRNAs in the testes from *Mov10l1* ^fl/-^ Neurog3-Cre mice. Moreover, the abundance of pachytene piRNAs was sharply reduced in *Mov10l1* ^fl/-^ Hspa2-Cre testes (Figure 2D). In addition, Northern blotting showed that individual pachytene piRNAs (piR1, piR2, and piR3) were absent in testes from *Mov10l1* ^fl/-^ Neurog3-Cre mice and dramatically reduced in abundance in testes from *Mov10l1* ^fl/-^ Hspa2-Cre mice (Figure 3). As expected, the abundance of individual pachytene piRNAs was not affected in the testes from *Mov10l1* ^fl/-^ Prm-Cre mice (Figure 3). Therefore, MOV10L1 function is essential for the biogenesis of all pachytene piRNAs.

### Production of Pre-Pachytene piRNAs Is Not Affected in *Mov10l1* ^fl/-^ Neurog3-Cre Testes

Pre-pachytene piRNAs are present in mitotic germ cells such as spermatogonia (Figure 1A). Because Neurog3-Cre initiated the disruption of *Mov10l1* at post-natal day 9, we anticipated that the production of pre-pachytene piRNAs would not be affected in *Mov10l1* ^fl/-^ Neurog3-Cre testes. To test this hypothesis, we performed immunoprecipitation of postnatal day 10 testis lysates with anti-MILI antibodies. Postnatal day 10 testes do not contain pachytene spermatocytes, and express only MILI but not other Piwi proteins (Figure 1A). Consequently, all MILI-bound piRNAs in postnatal day 10 testes are pre-pachytene piRNAs [10]. We found that pre-pachytene piRNAs were present in postnatal day 10 *Mov10l1* ^fl/-^ Neurog3-Cre testes (Figure 2E). Furthermore, Northern blot analysis showed that abundance of a specific pre-pachytene piRNA was not reduced in adult testes from *Mov10l1* mutant mice, regardless of whether deletion had been mediated by Neurog3-Cre, Hspa2-Cre, or Prm-Cre (Figure 3). These data demonstrate that pre-pachytene piRNA production is not affected in the *Mov10l1* conditional mutant testes.

### Polar Conglomeration of piRNA Pathway Components with Mitochondria in Pachytene piRNA-Deficient Spermatocytes

**Figure 4:**
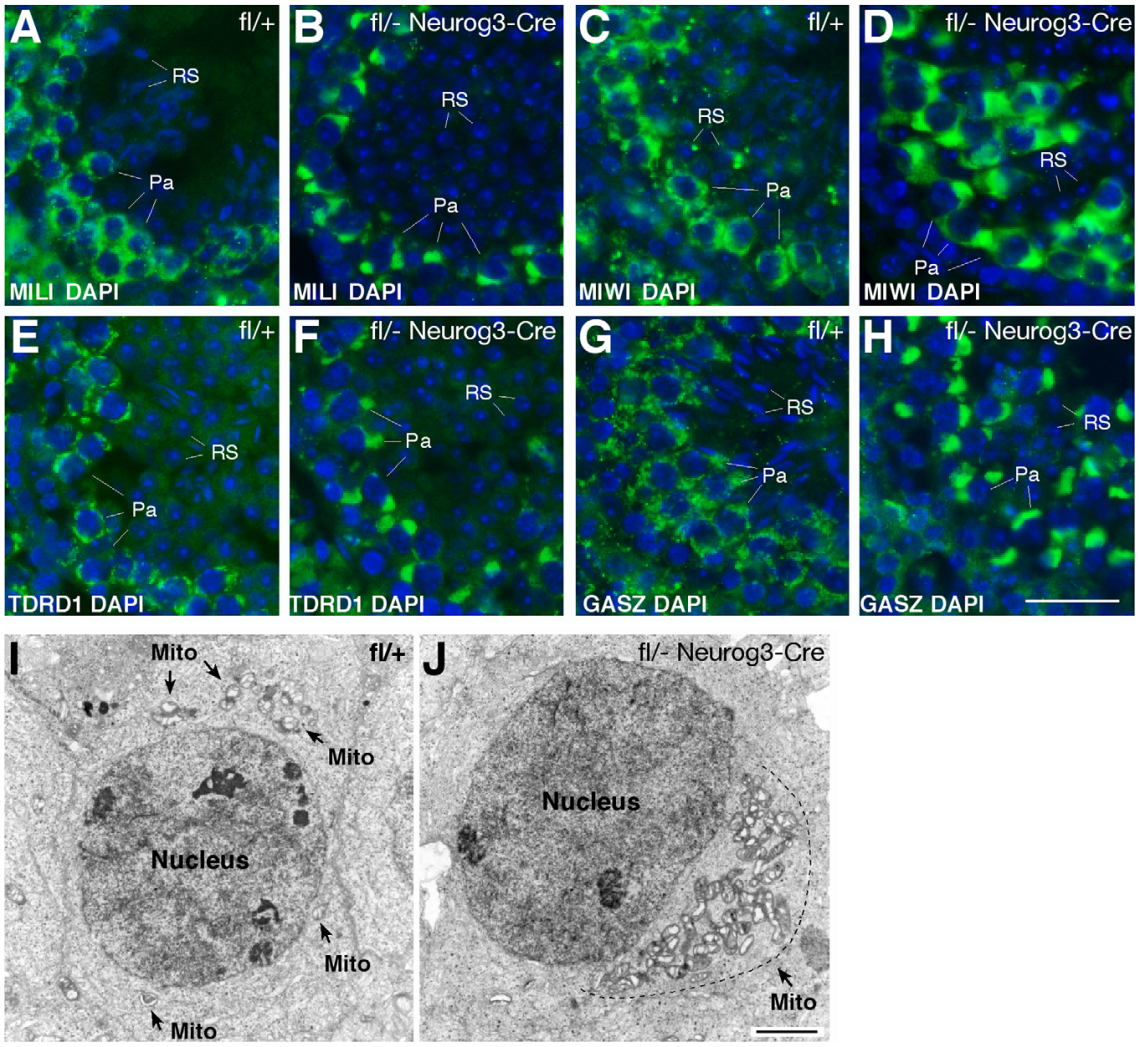
Polar conglomerate of piRNA pathway protein components and mitochondria in *Mov10l1*-deficient pachytene spermatocytes. Adult testis sections were immunostained with antibodies (in green) against MILI (A, B), MIWI (C, D), TDRD1 (E, F), and GASZ (G, H). DAPI staining of DNA is shown in blue. (I) EM analysis of a wild-type pachytene spermatocyte. Note the random distribution of mitochondria (indicated by arrows). (J) EM image of a *Mov10l1*-deficient pachytene spermatocyte with a single mitochondrial cluster. Mito, mitochondria. Scale bars: A–H, 25 µm; I–J, 2 µm.

We next examined the consequences of the loss of pachytene piRNAs on the localization of piRNA pathway components such as MILI, MIWI, TDRD1, and GASZ. In wild-type pachytene spermatocytes, these proteins localize to cytoplasmic nuage granules (also called inter-mitochondrial cement) (Figure 4A, 4C, 4E, 4G) [7], [8], [37], [44]. Strikingly, in *Mov10l1*-deficient pachytene spermatocytes, these four proteins congregated to one extremely large novel perinuclear polar “granule” (Figure 4B, 4D, 4F, and 4H). Further analyses revealed immunoreactivity of the polar granule to a cocktail of antibodies against mitochondrial proteins (OXPHOS), demonstrating co-localization of mitochondria with MILI in polar granules of *Mov10l1*-deficient pachytene spermatocytes (Figure S4). Electron microscopy (EM) analysis confirmed that in *Mov10l1*-deficient pachytene spermatocytes, mitochondria form a single cluster (Figure 4J), in contrast to their random distribution in wild-type pachytene cells (Figure 4I). Consistent with a recently described role for MitoPLD, a mitochondrial surface protein, in the piRNA pathway [39], [40], these data strongly suggest a novel but yet unknown role for mitochondria in the biogenesis of pachytene piRNAs and/or a function for pachytene piRNAs in the cytoplasmic organization and distribution of mitochondria and piRNA pathway protein components.

We next examined the status of chromatoid bodies, which are large and dynamic ribonucleoprotein aggregates prominent in haploid spermatids. Chromatoid bodies contain various RNA regulatory proteins as well as piRNA pathway components, but their precise function remains unclear [45]. Wild-type round spermatids contained one prominent chromatoid body, visualized by EM as a multi-lobular electron-dense nuage (Figure S5). In *Mov10l1*-deficient spermatids, however, the chromatoid body was fragmented (Figure S5). A similar fragmentation of chromatoid bodies has been observed in other mouse mutants of RNA processing pathway proteins with male infertility phenotype, implying importance of their structural integrity (*Miwi*, *Tdrd5*, and *Tdrd6*) [7], [18], [46], [47].

### Severe DNA Damage in piRNA-Deficient Round Spermatids

**Figure 5:**
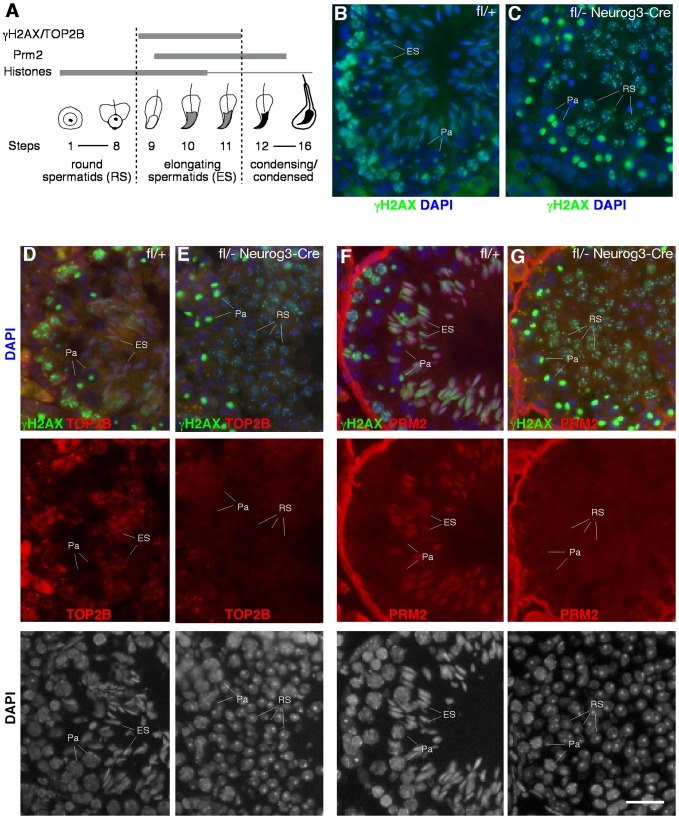
Massive DNA damage in piRNA-deficient round spermatids. (A) Schematic representation of the expression of several proteins involved in chromatin remodeling during mouse spermiogenesis. (B) Phosphorylation of histone H2AX during normal spermatogenesis. Note the presence of γH2AX in three distinct types of germ cells: leptotene/zygotene spermatocytes (due to meiotic recombination), pachytene spermatocytes (XY body only, due to sex chromosome silencing), and elongating spermatids (due to chromatin remodeling). (C) Presence of γH2AX in round spermatids from *Mov10l1^fl^* ^/-^ Neurog3-Cre testes. (D, E) Double staining of γH2AX and TOP2B in seminiferous tubules from wild-type and *Mov10l1^fl^* ^/-^ Neurog3-Cre testes. (F, G) Double staining of γH2AX and PRM2 in seminiferous tubules from wild-type and *Mov10l1^fl^* ^/-^ Neurog3-Cre testes. Red channels and DAPI staining are also shown in separate panels (D–G). Pa, pachytene spermatocytes; RS, round spermatids; ES, elongating spermatids. Scale bar, 25 µm.

**Figure 6:**
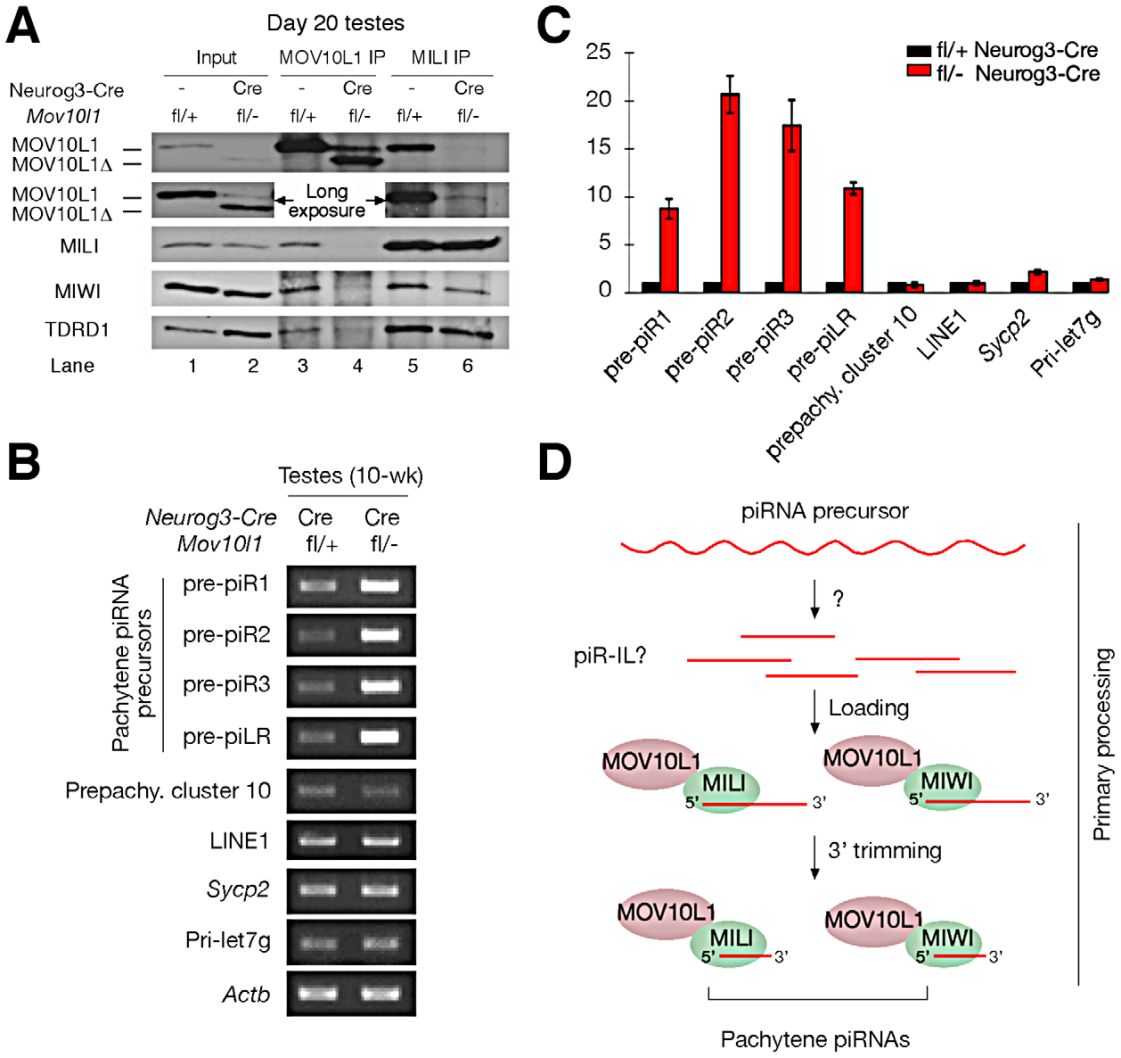
MOV10L1 is essential for the primary processing of pachytene piRNA precursors. (A) The RNA helicase domain in MOV10L1 is required for interaction with MILI and MIWI. Input was 1/50^th^ of extracts used for IP. TDRD1 is complexed with MILI and MOV10L1 and thus served as a positive control for immunoprecipitation. The same results were obtained from the repeat of IP experiments using independent samples. (B) Blockade of pachytene piRNA precursor processing in *Mov10l1* ^fl/-^ Neurog3-Cre testes. Total RNAs were pre-treated with DNase I. RT-PCR produced no products in controls without reverse transcriptase (data not shown). PCR primers and product sizes are listed in Table S2. (C) Quantitative RT-PCR analysis of piRNA precursor transcripts in adult testes. Numbers on the vertical axis represent fold increase of levels in mutant testis compared to levels in wild-type testes defined as 1. (D) Proposed model for the essential role of MOV10L1 in the primary processing of pachytene piRNAs. Single strand RNAs are transcribed from the piRNA clusters and are digested by an unknown nuclease into putative piRNA intermediate like molecules (piR ILs). Such piR ILs have been reported in *Drosophila* but not yet in mammals [20]. MOV10L1 and Piwi proteins select and bind to piR ILs for further processing into mature primary piRNAs through 3′ trimming [21].

The introduction of DNA double strand breaks (DSBs) into the germ cell genome takes place as part of the chromatin remodeling process occurring at the elongating spermatid stage (Figure 5A). This chromatin remodeling process is initiated by the replacement of canonical histones first with transition proteins and eventually by protamines. Concurrently, nucleosomal DNA supercoils must be resolved, presumably by topoisomerase IIB (TOP2B). TOP2B generates DNA double-strand breaks (DSBs), relaxes supercoils, and subsequently religates DNA ends [48]. DSBs trigger a DNA damage response, resulting in the phosphorylation of histone H2AX (γH2AX). In wild-type testis, histone H2AX phosphorylation is therefore detectable in several germ cell stages that undergo changes in their chromatin configuration, including elongating spermatids (Figure 5B), but it is absent from round spermatids. Intriguingly, round spermatids from *Mov10l1* ^fl/-^ Neurog3-Cre testes exhibit a high degree of DNA damage visualized by γH2AX (Figure 5C). This could be due to a developmental progression of piRNA-deficient round spermatids to the “elongating” spermatid stage without apparent morphological change. However, the absence of both TOP2B and PRM2 (protamine 2) in γH2AX-positive round spermatids from *Mov10l1* mutant testes indicated that these round spermatids were not undergoing chromatin remodeling, excluding that γH2AX-positivity was due to TOP2B activity (Figure 5E, 5G). Secondly, DNA damage might be induced by de-repressed transposable elements active in piRNA-deficient round spermatids. Genetic studies have shown that the piRNA pathway is required for silencing of retrotransposons such as LINE1 and IAP in pre-pachytene germ cells [19]. However, quantitative RT-PCR analysis revealed no de-repression of LINE1 (Figure 6B, 6C) or IAP in *Mov10l1* ^fl/-^ Neurog3-Cre testes, confirmed by immunofluorescent analyses of testis sections with anti-LINE1 and anti-IAP antibodies (Figure S6). Therefore, pachytene piRNAs are not required for silencing of LINE1 and IAP retrotransposons, although we cannot rule out the possibility that other transposable elements might be de-repressed in *Mov10l1*-deficient round spermatids. Notably, we did not observe γH2AX foci in round spermatids from *Rnf17*-deficient mice, in which spermatogenesis is also arrested at the round spermatid stage [49] but piRNA biogenesis does not appear to be severely affected (data not shown). These results suggest that the DSBs observed in round spermatids from *Mov10l1^fl^* ^/-^ Neurog3-Cre testes and *Mov10l1* ^fl/-^ Hspa2-Cre testes are not a direct consequence of their developmental arrest. Rather, these observations suggest that the piRNA pathway, specifically MOV10L1 and pachytene piRNAs, play a yet undefined role in maintaining genome integrity in post-meiotic round spermatids.

### The MOV10L1 RNA Helicase Domain Is Required for Its Interaction with Piwi Proteins

We previously found that MOV10L1 interacts with all three Piwi proteins (MILI, MIWI, and MIWI2) [22]. The low abundance of MOV10L1Δ in both *Mov10l1^−/−^* (ubiquitous null mutant) testes [22] and adult *Mov10l1* conditional mutant testes (Figure S2A) precluded co-immunoprecipitation (IP) experiments to ascertain if deletion of the helicase domain affected interaction of the truncated protein with Piwi proteins in vivo. However, a peak expression of MOV10L1Δ protein in post-natal day 20 *Mov10l1* ^fl/-^ Neurog3-Cre testes, with a level highly exceeding that of the remaining wild-type MOV10L1 (Figure S2A), allowed us to perform co-immunoprecipitation of day 20 testicular extracts with anti-MILI and anti- MOV10L1 antibodies (Figure 6A). While wild-type MOV10L1 (due to the lack of Neurog3-Cre expression in spermatogonia) could be detected as a very faint band in the MILI immunoprecipitate as expected, the much more abundant MOV10L1Δ was absent (Figure 6A, Lane 6). Notably, MILI was not detectable in the MOV10L1/MOV10L1Δ immunoprecipitate, and the level of MIWI was extremely low (Figure 6A, Lane 4). These results suggest that, apart from its putative enzymatic activity, the RNA helicase domain of MOV10L1 is also essential for its association with MILI and MIWI, and that piRNA production could be affected by disruption of the MOV10L1-Piwi interactions.

### Accumulation of Pachytene piRNA Precursors in *Mov10l1* Mutant Testes

Pachytene piRNAs are derived from only one strand of genomic clusters [11], [13]–[16], prompting the hypothesis that a single long primary piRNA transcript is made from each cluster and is cleaved into intermediate RNAs by an unknown Dicer-independent mechanism [5], [20], [21]. Due to their large size and low abundance, detection of these precursor transcripts requires RT-PCR analysis, with the exception of a ∼10 kb piLR (piRNA like small RNA) transcript that can be visualized on Northern blots of testicular extracts [50]. As the depletion of pachytene piRNAs in *Mov10l1* ^fl/-^ Neurog3-Cre testes may be due to a blockade of pachytene piRNA precursor processing, we examined the abundance of precursors of four pachytene piRNAs (piR1, piR2, piR3, and piLR) by RT-PCR assays (Figure S7). All four precursors accumulated substantially in *Mov10l1* ^fl/-^ Neurog3-Cre testes, at 8 to 20 fold increased levels (Figure 6B, 6C). As expected, abundance of the pre-pachytene piRNA precursor (cluster 10) [10] and the miRNA precursor Pri-let7g remained constant (Figure 6B, 6C). These data suggest that MOV10L1 is required for the primary processing of precursor transcripts and thus plays an essential role in the early steps of the piRNA biogenesis pathway, i.e. primary processing and loading onto Piwi proteins (Figure 6D).

## Discussion

We have identified MOV10L1 as the only factor known to date that is required for the production of all pachytene piRNAs in mouse. As the biogenesis of pachytene piRNAs only involves the primary processing pathway, our conditional *Mov10l1* mutants provide a unique opportunity to delineate this enigmatic component of piRNA biogenesis in mammalian species. Presumably, long piRNA precursor transcripts are first cleaved into intermediate molecules, and then processed into mature piRNAs (Figure 6D). Observations that the *Drosophila* Armi-Piwi-Yb complex is associated with a population of 25–70 nt piRNA intermediate-like (piR-IL) molecules support this hypothesis [20]. Furthermore, recent biochemical studies using silkworm ovarian cell lysate have shown that intermediate piRNA molecules with 5′ U are specifically loaded onto Piwi proteins and then trimmed from the 3′end to generate mature piRNAs [21]. Here, we show that, in the mouse male germline, postnatal disruption of *Mov10l1* does not affect the expression of Piwi proteins (MILI and MIWI) but causes a complete loss of pachytene piRNAs, demonstrating that MOV10L1 functions upstream of Piwi proteins in the piRNA biogenesis pathway. Consistent with its homology to *Drosophila* Armi [25], [26], MOV10L1 is therefore a master regulator of piRNA biogenesis in mouse. This notion is further supported by the dramatic accumulation of pachytene piRNA precursors in the *Mov10l1* mutant testes. As MOV10L1 interacts with Piwi proteins, we postulate that MOV10L1 may facilitate the loading of intermediate piRNA molecules onto the Piwi proteins in mouse (Figure 6D).

In spermatocytes, proteins of the piRNA pathway such as MILI, MIWI, TDRD1, MAEL, and GASZ, localize to the nuage - inter-mitochondrial cement [7], [8], [36], [37], [44], [51]. However, the functional significance of the physical association of nuage with mitochondria in germ cells is poorly understood. MitoPLD, a mitochondrial signaling protein, is essential for nuage formation and piRNA production, suggesting an important role for mitochondria in these mechanisms [39], [40]. In this study, we find an unusual polar congregation of piRNA pathway proteins (such as MILI, MIWI, TDRD1, and GASZ). Similar to wild-type MOV10L1, truncated MOV10L1Δ is distributed diffusely through the cytoplasm of pachytene spermatocytes; therefore the polar coalescence of the other piRNA pathway components in MOV10L1-deficient pachytene cells is likely caused by the absence of pachytene piRNAs. However, as the association of Piwi-MOV10L1 is disrupted in the *Mov10l1* mutant, it is also possible that the localization of Piwi proteins and their interacting partners has become perturbed as a consequence of this disruption. The unusual polar congregation of piRNA pathway proteins with mitochondria in *Mov10l1* mutant spermatocytes suggests that MOV10L1 and/or pachytene piRNAs are essential for nuage formation and proper mitochondria distribution. Consistent with such a role, we find that the chromatoid body, a prominent nuage in spermatids, is fragmented in pachytene piRNA-deficient mutant cells. This previously unknown role in organelle distribution shows that pachytene piRNAs are intricately integrated in the inter-dependent relationships among piRNA production, nuage formation, and mitochondria organization that are essential for male germ cell maturation.

A recent study has shown that MIWI is an RNA-guided RNase with slicer activity that directly cleaves transcripts of the LINE1 retrotransposon [18]. *Miwi*-deficient and *Miwi* ^ADH^ (slicer inactive) mutant testes, in which MIWI is either absent or lacks slicer activity, exhibit substantial accumulation of LINE1 transcripts and protein. In *Mov10l1* ^fl/-^ Neurog3-Cre testes, however, LINE1 RNA levels are not affected. One possible explanation for these differential effects on LINE1 abundance could be that, in *Mov10l1* ^fl/-^ Neurog3-Cre testes, MIWI is catalytically intact and may function as a slicer through pachytene piRNA-independent mechanisms. Moreover, MIWI directly binds to spermiogenic mRNAs, independent of piRNAs [17].

Although previous genetic studies of piRNA pathway mutants show that perturbation of pre-pachytene piRNAs causes meiotic arrest and de-repression of LINE1 and IAP retrotransposons, the functions of pachytene piRNAs have remained elusive. Our study on the role of *Mov10l1* and the piRNA pathway during later stages of meiosis and spermiogenesis demonstrates that pachytene piRNAs fulfill distinct and essential functions during post-meiotic stages of male germ cell development. Most importantly, the massive DNA damage observed in piRNA-deficient round spermatids in the absence of de-repression of LINE1 and IAP transposable elements suggests that the integrity of the post-meiotic germ cell genome remains highly prone to damage, and that pachytene piRNAs fulfill a protective role at this stage by yet undefined mechanisms.

## Materials and Methods

### Ethics Statement

Mice were maintained and used for experimentation according to the guidelines of the Institutional Animal Care and Use Committee of the University of Pennsylvania.

### Mice and Antibodies

Neurog3-Cre, Hspa2-Cre, and Prm-Cre mice were purchased from the Jackson Laboratory (Stock numbers: Neurog3-Cre, 006333; Hspa2-Cre, 008870; Prm-Cre, 003328). *Mov10l1* ^fl/fl^ mice were generated previously [22]. Genotyping for *Mov10l1* and Cre alleles was performed separately on genomic DNA isolated from tails. The anti-MOV10L1 antibody was generated previously [22]. Other antibodies used were MILI (Abcam), MIWI (Abcam, or gifts from R. Pillai), GASZ (M. M. Matzuk), LINE1 ORF1p (S. L. Martin), IAP (B. R. Cullen), TDRD1 (S. Chuma), TOP2B (Santa Cruz Biotechnology), PRM2 (SHAL), and ACTB (Sigma-Aldrich).

### Immunoprecipitation and Detection of piRNAs

Mouse testicular extract preparation, immunoprecipitation, and 5′ end-labeling of piRNAs were performed as described previously [22]. Antibodies were described previously [22].

### Northern Blot Analysis of piRNAs

Northern blot analyses were performed as previously described with modifications [14]. Total RNAs were isolated from mouse testes using Trizol reagent, separated by 15% denaturing polyacrylamide gel, and electro-blotted onto GeneScreen Plus hybridization membrane. Membranes were UV crosslinked and hybridized with ^32^P end-labeled oligonucleotide probes in Ultrahyb Oligo Buffer (Ambion Cat#8663) at 42°C. Probes for detecting pachytene piRNAs, a pre-pachytene piRNA, or microRNA were perfectly complementary to their sequences: probe-piR1: AAAGCTATCTGAGCACCTGTGTTCATGTCA; probe-piR2: ACCAGCAGACACCGTCGTATGCATCACACA; probe-piR3: ACCACTAAACATTTAGATGCCACTCTCA; probe-let7g: TACTGTACAAACTACTACCTCA; pre-pachytene piRNA probe (derived from sense SINE B1): 5′-TGGCTGTCCTGGAACTCACTYTGT [10]. After hybridization, membranes were washed three times at 42°C in 2×SSC buffer containing 0.5% SDS, or stripped by boiling in 0.1×SSC containing 0.1% SDS. Membranes were exposed to a phosphor imager screen for autoradiography.

### Histological, Immunofluorescence, and Electron Microscopy (EM) Analyses

For histology, testes were fixed in Bouin's solution overnight, processed, sectioned, and stained with hematoxylin and eosin. Immunofluorescence was performed on frozen sections of testes fixed in 4% paraformaldehyde as previously described [52]. EM followed a standard protocol used at the Electron Microscopy Resource Laboratory of the University of Pennsylvania.

### Expression Analysis of piRNA Precursor Transcripts

PCR primers for piRNA precursor transcripts were chosen from genomic clusters to which each piRNA was mapped [10], [14], [50]. PCR primers and PCR product sizes are listed in Table S2.

## Supporting Information

Figure S1 Breeding schemes for *Mov10l1* conditional mutant mice. The breeding scheme for generating *Mov10l1* ^fl/-^ Neurog3-Cre mice is shown as an example. The same strategy was applied to generate *Mov10l1* ^fl/-^ Hspa2-Cre and *Mov10l1* ^fl/-^ Prm-Cre mice. Genotypes were identified by PCR analysis of tail genomic DNA.
(TIF)

Figure S2 Developmental stage-specific inactivation of *Mov10l1* in the male germline. (A) Western blot analysis of *Mov10l1* ^fl/-^ Neurog3-Cre testicular extracts. Conditional disruption of MOV10L1 is associated with a decrease in the abundance of the full-length MOV10L1 and appearance of truncated MOV10L1Δ. Genotypes indicated were identified using tail genomic DNA. (B) Western blot analysis of *Mov10l1* ^fl/-^ Hspa2-Cre testes.
(TIF)

Figure S3 Histological analysis of testes from adult wild-type (A) and *Mov10l1* ^fl/-^ Prm-Cre (B) mice. Testis sections were stained with H&E as described in the Materials and Methods. In testes from *Mov10l1* ^fl/-^ Prm-Cre (B) mice, spermatogenesis appears to be normal. Abbreviations: Pa, pachytene spermatocytes; RS, round spermatids; ES, elongated spermatids. Scale bar, 25 µm.
(TIF)

Figure S4 Co-clustering of MILI with mitochondria in *Mov10l1*-deficient pachytene spermatocytes. Testis sections from adult wild-type (A) and *Mov10l1* ^fl/-^ Neurog3-Cre mice (B) were immunostained with anti-MILI antibody and a mixture of five monoclonal antibodies against mitochondrial components (OXPHOS cocktail, Mito Sciences). In wild-type pachytene spermatocytes, MILI and mitochondria were mostly dispersed throughout the cytoplasm (A), however, mitochondria clustered to the same polar cytoplasmic location as MILI in the *Mov10l1*-deficient pachytene spermatocytes (representative pachytene cells were indicated by arrows in B). Co-localization in the pachytene cell (indicated by a large arrow) is shown in high magnification in the inset. Scale bar, 50 µm.
(TIF)

Figure S5 Fragmentation of chromatoid bodies in *Mov10l1*-deficient round spermatids. EM analysis of spermatids from wild-type and *Mov10l1* ^fl/-^ Neurog3-Cre testes showed multi-lobular and prominent chromatoid bodies in wild-type round spermatids (A, B), but fragmented and much less prominent chromatoid bodies in *Mov10l1*-deficient round spermatids (C, D). Scale bar, 2 µm.
(TIF)

Figure S6 Immunofluorescence analysis of LINE1 and IAP in testes from adult wild-type (A, D), *Mov10l1* ^fl/-^ Neurog3-Cre (B, E), and *Mov10l1* ^−/−^ (C, F) mice. Leydig cells reside in the interstitium and emit a strong autofluorescence signal. In *Mov10l1* ^−/−^ (ubiquitous knockout) testes, LINE1 (C) and IAP (F) are de-repressed in spermatocytes and spermatogonia, respectively [22], providing a positive control . In contrast, LINE1 (B) and IAP (E) are not de-repressed in *Mov10l1* ^fl/-^ Neurog3-Cre testes. Scale bar, 50 µm.
(TIF)

Figure S7 Detection of pachytene and pre-pachytene piRNA precursor transcripts by semi-quantitative RT-PCR. In postnatal day 10 testes, the most advanced germ cells are leptotene/zygotene spermatocytes. Pachytene spermatocytes appear first in day 14 testes. *Mov10l1*-deficient (ubiquitous knockout, *Mov10l1* ^−/−^) testes exhibit meiotic arrest at the zygotene stage and thus lack pachytene spermatocytes and spermatids [22]. In wild-type testes, the cluster 10 pre-pachytene piRNA precursor is abundantly expressed at day 10, while pachytene precursors are first detectable at day 14. Pachytene piRNA precursors are not detectable in *Mov10l1* ^−/−^ testes. *Actb* served as a loading control.
(TIF)

Table S1 Testis weight, sperm production, and fertility of wild type and *Mov10l1* ^fl/-^ Prm-Cre (mutant) male mice.
(DOC)

Table S2 PCR primers for semi-quantitative RT-PCR and qPCR assays.
(DOC)

## References

[1] Siomi MC, Sato K, Pezic D, Aravin AA (2011) PIWI-interacting small RNAs: The vanguard of genome defence. Nat Rev Mol Cell Biol 12: 246–258. 21427766 10.1038/nrm3089

[2] Pillai RS, Chuma S (2012) piRNAs and their involvement in male germline development in mice. Dev Growth Differ 10.1111/j.1440-169X.2011.01320.x 22221002

[3] Aravin AA, Naumova NM, Tulin AV, Vagin VV, Rozovsky YM, et al (2001) Double-stranded RNA-mediated silencing of genomic tandem repeats and transposable elements in the D. melanogaster germline. Curr Biol 11: 1017–1027. 11470406 10.1016/s0960-9822(01)00299-8

[4] Brennecke J, Aravin AA, Stark A, Dus M, Kellis M, et al (2007) Discrete small RNA-generating loci as master regulators of transposon activity in drosophila. Cell 128: 1089–1103. 17346786 10.1016/j.cell.2007.01.043

[5] Haase AD, Fenoglio S, Muerdter F, Guzzardo PM, Czech B, et al (2010) Probing the initiation and effector phases of the somatic piRNA pathway in drosophila. Genes Dev 24: 2499–2504. 20966049 10.1101/gad.1968110 PMC2975926

[6] Aravin AA, Sachidanandam R, Bourc'his D, Schaefer C, Pezic D, et al (2008) A piRNA pathway primed by individual transposons is linked to de novo DNA methylation in mice. Mol Cell 31: 785–799. 18922463 10.1016/j.molcel.2008.09.003 PMC2730041

[7] Deng W, Lin H (2002) Miwi, a murine homolog of piwi, encodes a cytoplasmic protein essential for spermatogenesis. Dev Cell 2: 819–830. 12062093 10.1016/s1534-5807(02)00165-x

[8] Kuramochi-Miyagawa S, Kimura T, Ijiri TW, Isobe T, Asada N, et al (2004) Mili, a mammalian member of piwi family gene, is essential for spermatogenesis. Development 131: 839–849. 14736746 10.1242/dev.00973

[9] Kuramochi-Miyagawa S, Watanabe T, Gotoh K, Totoki Y, Toyoda A, et al (2008) DNA methylation of retrotransposon genes is regulated by piwi family members MILI and MIWI2 in murine fetal testes. Genes Dev 22: 908–917. 18381894 10.1101/gad.1640708 PMC2279202

[10] Aravin AA, Sachidanandam R, Girard A, Fejes-Toth K, Hannon GJ (2007) Developmentally regulated piRNA clusters implicate MILI in transposon control. Science 316: 744–747. 17446352 10.1126/science.1142612

[11] Gan H, Lin X, Zhang Z, Zhang W, Liao S, et al (2011) piRNA profiling during specific stages of mouse spermatogenesis. RNA 17: 1191–1203. 21602304 10.1261/rna.2648411 PMC3138557

[12] Watanabe T, Takeda A, Tsukiyama T, Mise K, Okuno T, et al (2006) Identification and characterization of two novel classes of small RNAs in the mouse germline: Retrotransposon-derived siRNAs in oocytes and germline small RNAs in testes. Genes Dev 20: 1732–1743. 16766679 10.1101/gad.1425706 PMC1522070

[13] Lau NC, Seto AG, Kim J, Kuramochi-Miyagawa S, Nakano T, et al (2006) Characterization of the piRNA complex from rat testes. Science 313: 363–367. 16778019 10.1126/science.1130164

[14] Girard A, Sachidanandam R, Hannon GJ, Carmell MA (2006) A germline-specific class of small RNAs binds mammalian piwi proteins. Nature 442: 199–202. 16751776 10.1038/nature04917

[15] Grivna ST, Beyret E, Wang Z, Lin H (2006) A novel class of small RNAs in mouse spermatogenic cells. Genes Dev 20: 1709–1714. 16766680 10.1101/gad.1434406 PMC1522066

[16] Aravin A, Gaidatzis D, Pfeffer S, Lagos-Quintana M, Landgraf P, et al (2006) A novel class of small RNAs bind to MILI protein in mouse testes. Nature 442: 203–207. 16751777 10.1038/nature04916

[17] Vourekas A, Zheng Q, Alexiou P, Maragkakis M, Kirino Y, et al (2012) Mili and miwi target RNA repertoire reveals piRNA biogenesis and function of miwi in spermiogenesis. Nat Struct Mol Biol 19: 773–781. 22842725 10.1038/nsmb.2347 PMC3414646

[18] Reuter M, Berninger P, Chuma S, Shah H, Hosokawa M, et al (2011) Miwi catalysis is required for piRNA amplification-independent LINE1 transposon silencing. Nature 480: 264–267. 22121019 10.1038/nature10672

[19] De Fazio S, Bartonicek N, Di Giacomo M, Abreu-Goodger C, Sankar A, et al (2011) The endonuclease activity of mili fuels piRNA amplification that silences LINE1 elements. Nature 480: 259–263. 22020280 10.1038/nature10547

[20] Saito K, Ishizu H, Komai M, Kotani H, Kawamura Y, et al (2010) Roles for the yb body components armitage and yb in primary piRNA biogenesis in drosophila. Genes Dev 24: 2493–2498. 20966047 10.1101/gad.1989510 PMC2975925

[21] Kawaoka S, Izumi N, Katsuma S, Tomari Y (2011) 3′ end formation of PIWI-interacting RNAs in vitro. Mol Cell 43: 1015–1022. 21925389 10.1016/j.molcel.2011.07.029

[22] Zheng K, Xiol J, Reuter M, Eckardt S, Leu NA, et al (2010) Mouse MOV10L1 associates with piwi proteins and is an essential component of the piwi-interacting RNA (piRNA) pathway. Proc Natl Acad Sci U S A 107: 11841–11846. 20534472 10.1073/pnas.1003953107 PMC2900664

[23] Frost RJ, Hamra FK, Richardson JA, Qi X, Bassel-Duby R, et al (2010) MOV10L1 is necessary for protection of spermatocytes against retrotransposons by piwi-interacting RNAs. Proc Natl Acad Sci U S A 107: 11847–11852. 20547853 10.1073/pnas.1007158107 PMC2900665

[24] Dalmay T, Horsefield R, Braunstein TH, Baulcombe DC (2001) SDE3 encodes an RNA helicase required for post-transcriptional gene silencing in arabidopsis. EMBO J 20: 2069–2078. 11296239 10.1093/emboj/20.8.2069 PMC125430

[25] Tomari Y, Du T, Haley B, Schwarz DS, Bennett R, et al (2004) RISC assembly defects in the drosophila RNAi mutant armitage. Cell 116: 831–841. 15035985 10.1016/s0092-8674(04)00218-1

[26] Cook HA, Koppetsch BS, Wu J, Theurkauf WE (2004) The drosophila SDE3 homolog armitage is required for oskar mRNA silencing and embryonic axis specification. Cell 116: 817–829. 15035984 10.1016/s0092-8674(04)00250-8

[27] Vagin VV, Sigova A, Li C, Seitz H, Gvozdev V, et al (2006) A distinct small RNA pathway silences selfish genetic elements in the germline. Science 313: 320–324. 16809489 10.1126/science.1129333

[28] Malone CD, Brennecke J, Dus M, Stark A, McCombie WR, et al (2009) Specialized piRNA pathways act in germline and somatic tissues of the drosophila ovary. Cell 137: 522–535. 19395010 10.1016/j.cell.2009.03.040 PMC2882632

[29] Olivieri D, Sykora MM, Sachidanandam R, Mechtler K, Brennecke J (2010) An in vivo RNAi assay identifies major genetic and cellular requirements for primary piRNA biogenesis in drosophila. EMBO J 29: 3301–3317. 20818334 10.1038/emboj.2010.212 PMC2957214

[30] Meister G, Landthaler M, Peters L, Chen PY, Urlaub H, et al (2005) Identification of novel argonaute-associated proteins. Curr Biol 15: 2149–2155. 16289642 10.1016/j.cub.2005.10.048

[31] Chendrimada TP, Finn KJ, Ji X, Baillat D, Gregory RI, et al (2007) MicroRNA silencing through RISC recruitment of eIF6. Nature 447: 823–828. 17507929 10.1038/nature05841

[32] Wang PJ, McCarrey JR, Yang F, Page DC (2001) An abundance of X-linked genes expressed in spermatogonia. Nat Genet 27: 422–426. 11279525 10.1038/86927

[33] Wang PJ, Page DC, McCarrey JR (2005) Differential expression of sex-linked and autosomal germ-cell-specific genes during spermatogenesis in the mouse. Hum Mol Genet 14: 2911–2918. 16118233 10.1093/hmg/ddi322 PMC1994333

[34] Carmell MA, Girard A, van de Kant HJ, Bourc'his D, Bestor TH, et al (2007) MIWI2 is essential for spermatogenesis and repression of transposons in the mouse male germline. Dev Cell 12: 503–514. 17395546 10.1016/j.devcel.2007.03.001

[35] Tanaka SS, Toyooka Y, Akasu R, Katoh-Fukui Y, Nakahara Y, et al (2000) The mouse homolog of drosophila vasa is required for the development of male germ cells. Genes Dev 14: 841–853. 10766740 PMC316497

[36] Soper SF, van der Heijden GW, Hardiman TC, Goodheart M, Martin SL, et al (2008) Mouse maelstrom, a component of nuage, is essential for spermatogenesis and transposon repression in meiosis. Dev Cell 15: 285–297. 18694567 10.1016/j.devcel.2008.05.015 PMC2546488

[37] Ma L, Buchold GM, Greenbaum MP, Roy A, Burns KH, et al (2009) GASZ is essential for male meiosis and suppression of retrotransposon expression in the male germline. PLoS Genet 5: e1000635 doi:10.1371/journal.pgen.1000635. 19730684 10.1371/journal.pgen.1000635 PMC2727916

[38] Shoji M, Tanaka T, Hosokawa M, Reuter M, Stark A, et al (2009) The TDRD9-MIWI2 complex is essential for piRNA-mediated retrotransposon silencing in the mouse male germline. Dev Cell 17: 775–787. 20059948 10.1016/j.devcel.2009.10.012

[39] Watanabe T, Chuma S, Yamamoto Y, Kuramochi-Miyagawa S, Totoki Y, et al (2011) MITOPLD is a mitochondrial protein essential for nuage formation and piRNA biogenesis in the mouse germline. Dev Cell 20: 364–375. 21397847 10.1016/j.devcel.2011.01.005 PMC3062204

[40] Huang H, Gao Q, Peng X, Choi SY, Sarma K, et al (2011) piRNA-associated germline nuage formation and spermatogenesis require MitoPLD profusogenic mitochondrial-surface lipid signaling. Dev Cell 20: 376–387. 21397848 10.1016/j.devcel.2011.01.004 PMC3061402

[41] Schonhoff SE, Giel-Moloney M, Leiter AB (2004) Neurogenin 3-expressing progenitor cells in the gastrointestinal tract differentiate into both endocrine and non-endocrine cell types. Dev Biol 270: 443–454. 15183725 10.1016/j.ydbio.2004.03.013

[42] Inselman AL, Nakamura N, Brown PR, Willis WD, Goulding EH, et al (2010) Heat shock protein 2 promoter drives cre expression in spermatocytes of transgenic mice. Genesis 48: 114–120. 20027617 10.1002/dvg.20588 PMC2909465

[43] O'Gorman S, Dagenais NA, Qian M, Marchuk Y (1997) Protamine-cre recombinase transgenes efficiently recombine target sequences in the male germ line of mice, but not in embryonic stem cells. Proc Natl Acad Sci U S A 94: 14602–14607. 9405659 10.1073/pnas.94.26.14602 PMC25067

[44] Chuma S, Hosokawa M, Kitamura K, Kasai S, Fujioka M, et al (2006) Tdrd1/Mtr-1, a tudor-related gene, is essential for male germ-cell differentiation and nuage/germinal granule formation in mice. Proc Natl Acad Sci U S A 103: 15894–15899. 17038506 10.1073/pnas.0601878103 PMC1635099

[45] Meikar O, Da Ros M, Korhonen H, Kotaja N (2011) Chromatoid body and small RNAs in male germ cells. Reproduction 142: 195–209. 21652638 10.1530/REP-11-0057

[46] Yabuta Y, Ohta H, Abe T, Kurimoto K, Chuma S, et al (2011) TDRD5 is required for retrotransposon silencing, chromatoid body assembly, and spermiogenesis in mice. J Cell Biol 192: 781–795. 21383078 10.1083/jcb.201009043 PMC3051809

[47] Vasileva A, Tiedau D, Firooznia A, Muller-Reichert T, Jessberger R (2009) Tdrd6 is required for spermiogenesis, chromatoid body architecture, and regulation of miRNA expression. Curr Biol 19: 630–639. 19345099 10.1016/j.cub.2009.02.047 PMC2719840

[48] Leduc F, Maquennehan V, Nkoma GB, Boissonneault G (2008) DNA damage response during chromatin remodeling in elongating spermatids of mice. Biol Reprod 78: 324–332. 18032420 10.1095/biolreprod.107.064162

[49] Pan J, Goodheart M, Chuma S, Nakatsuji N, Page DC, et al (2005) RNF17, a component of the mammalian germ cell nuage, is essential for spermiogenesis. Development 132: 4029–4039. 16093322 10.1242/dev.02003 PMC1994335

[50] Ro S, Park C, Song R, Nguyen D, Jin J, et al (2007) Cloning and expression profiling of testis-expressed piRNA-like RNAs. RNA 13: 1693–1702. 17698640 10.1261/rna.640307 PMC1986815

[51] Chuma S, Hosokawa M, Tanaka T, Nakatsuji N (2009) Ultrastructural characterization of spermatogenesis and its evolutionary conservation in the germline: Germinal granules in mammals. Mol Cell Endocrinol 306: 17–23. 19063939 10.1016/j.mce.2008.11.009

[52] Zheng K, Wu X, Kaestner KH, Wang PJ (2009) The pluripotency factor LIN28 marks undifferentiated spermatogonia in mouse. BMC Dev Biol 9: 38. 19563657 10.1186/1471-213X-9-38 PMC2719617

