# Supplementary figures and images for: Blockade of Pachytene piRNA Biogenesis Reveals a Novel Requirement for Maintaining Post-Meiotic Germline Genome Integrity
Source: PLoS Genet. 2012 Nov 15;8(11):e1003038. doi: 10.1371/journal.pgen.1003038 (PMC3499362; doi:10.1371/journal.pgen.1003038)

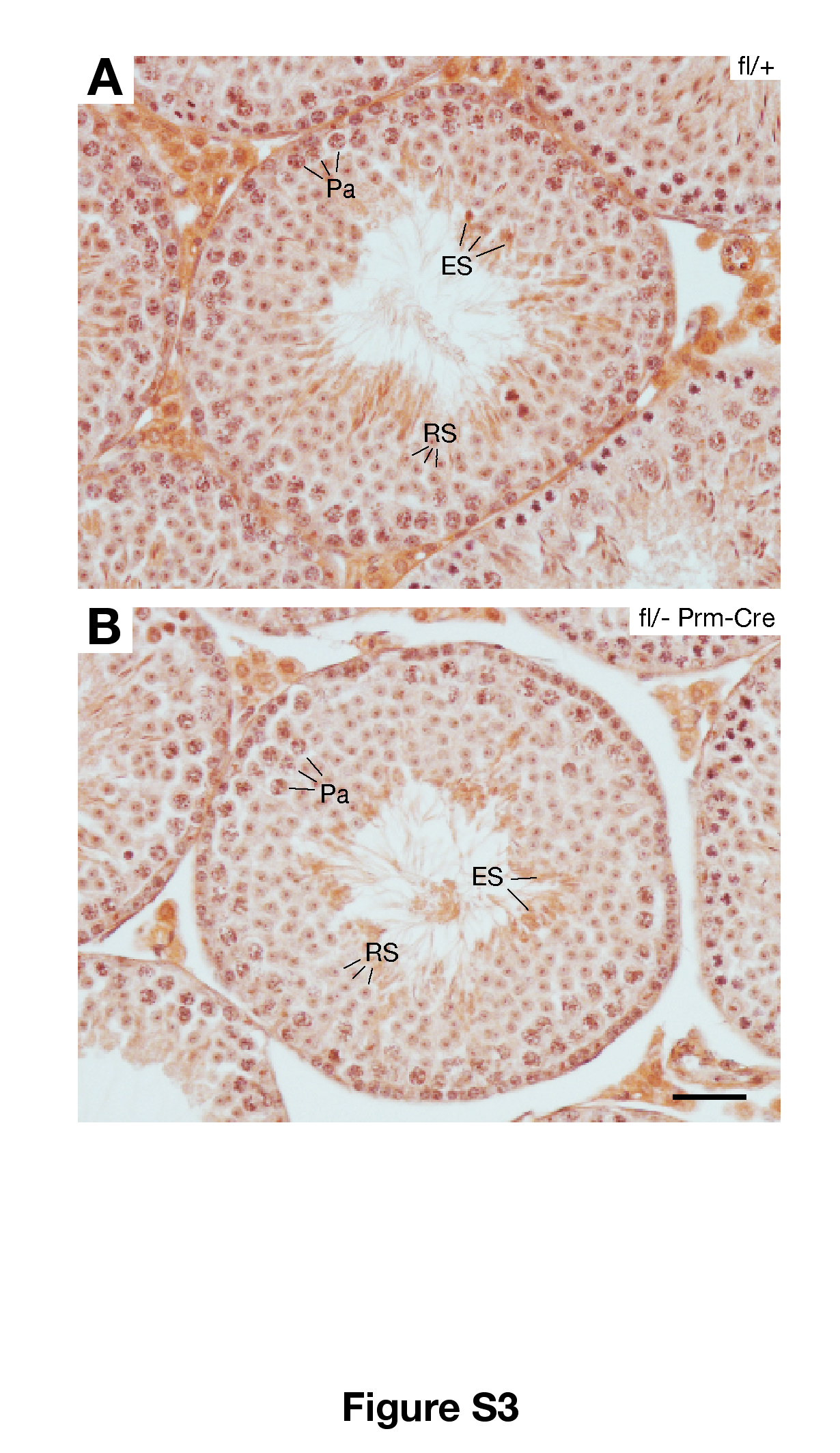

Supplement: Figure S3 — Histological analysis of testes from adult wild-type (A) and Mov10l1 fl/- Prm-Cre (B) mice. Testis sections were stained with H&E as described in the Materials and Methods. In testes from Mov10l1 fl/- Prm-Cre (B) mice, spermatogenesis appears to be normal. Abbreviations: Pa, pachytene spermatocytes; RS, round spermatids; ES, elongated spermatids. Scale bar, 25 µm. (TIF) [file pgen.1003038.s003.tif]

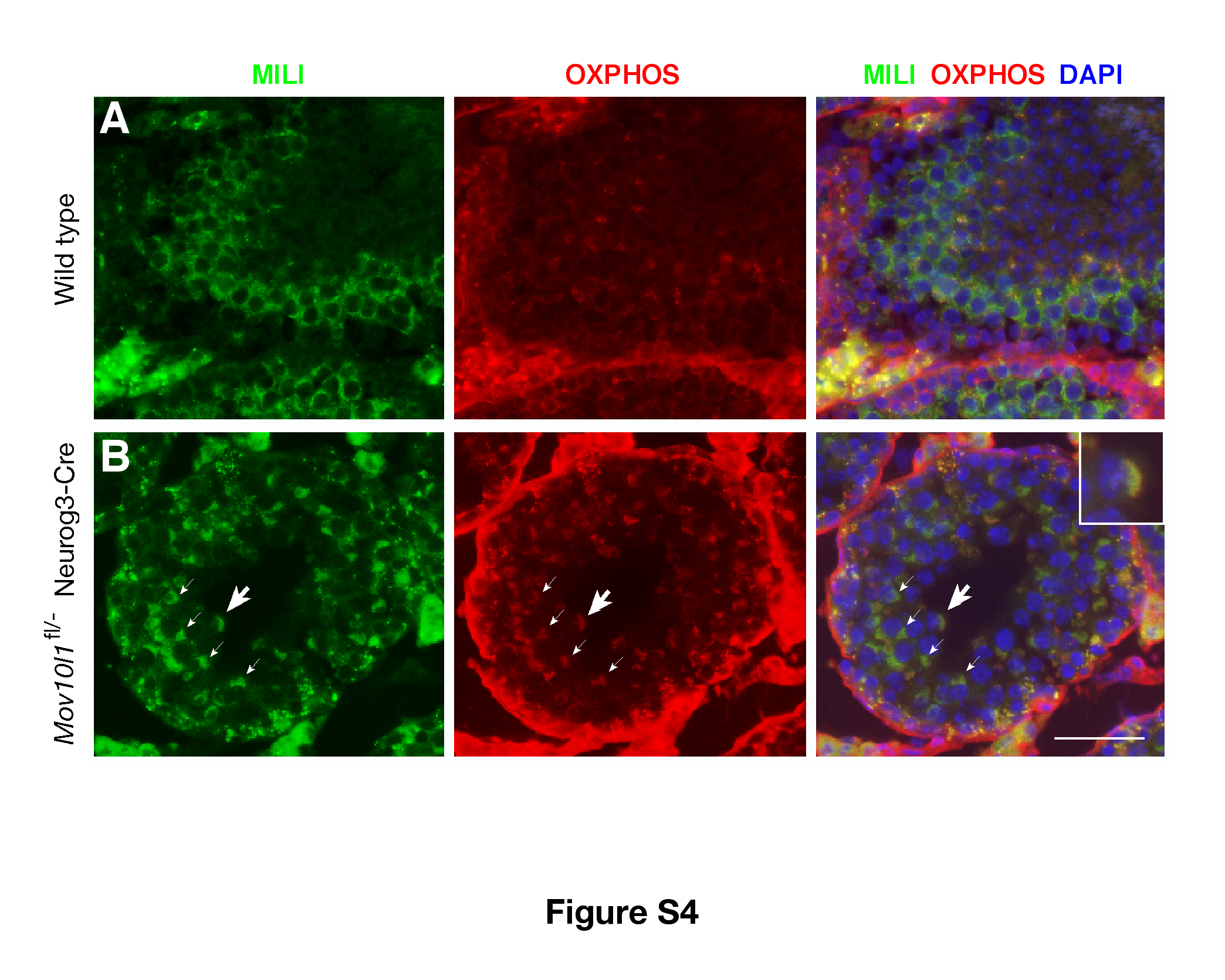

Supplement: Figure S4 — Co-clustering of MILI with mitochondria in Mov10l1-deficient pachytene spermatocytes. Testis sections from adult wild-type (A) and Mov10l1 fl/- Neurog3-Cre mice (B) were immunostained with anti-MILI antibody and a mixture of five monoclonal antibodies against mitochondrial components (OXPHOS cocktail, Mito Sciences). In wild-type pachytene spermatocytes, MILI and mitochondria were mostly dispersed throughout the cytoplasm (A), however, mitochondria clustered to the same polar cytoplasmic location as MILI in the Mov10l1-deficient pachytene spermatocytes (representative pachytene cells were indicated by arrows in B). Co-localization in the pachytene cell (indicated by a large arrow) is shown in high magnification in the inset. Scale bar, 50 µm. (TIF) [file pgen.1003038.s004.tif]

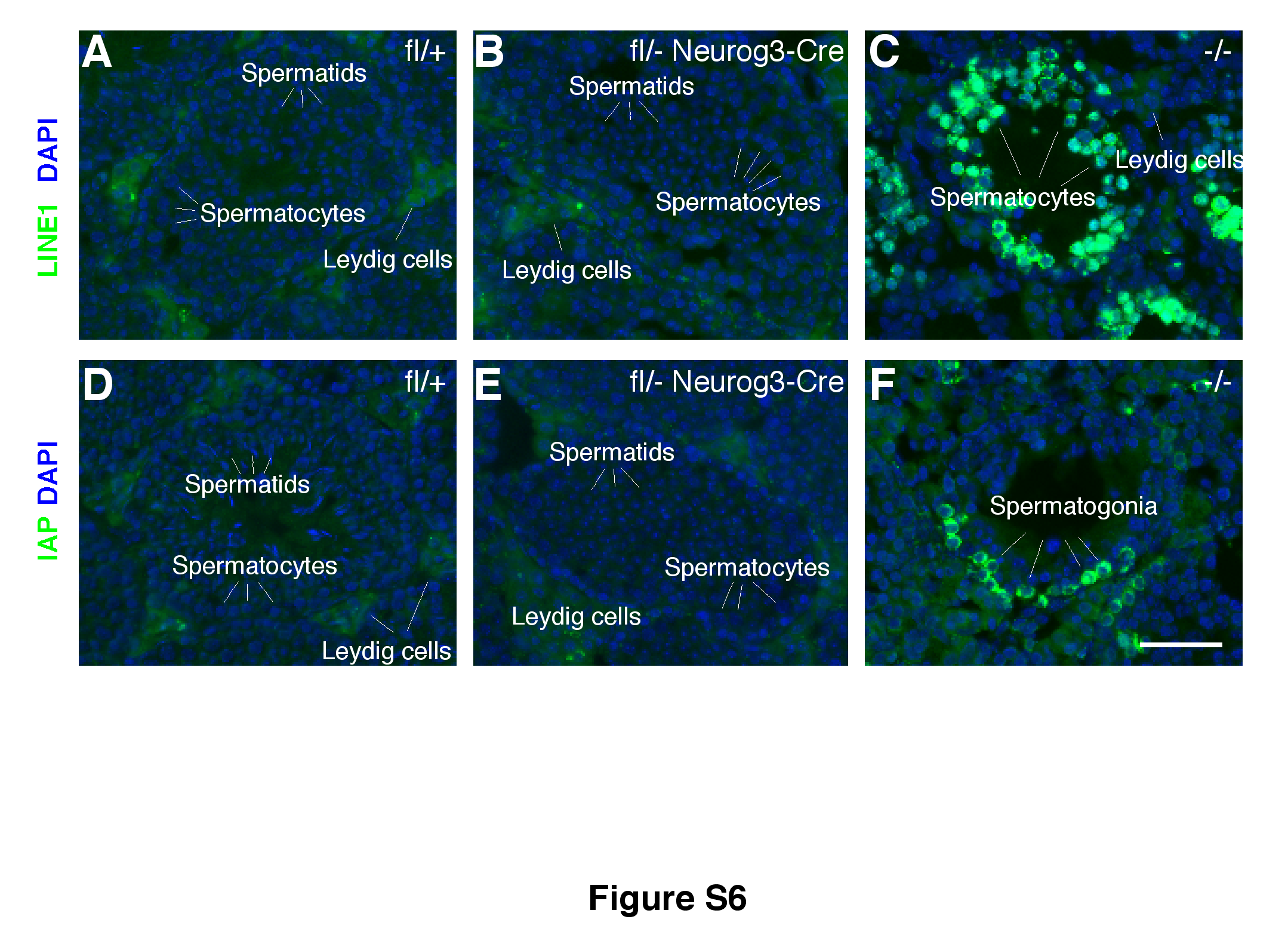

Supplement: Figure S6 — Immunofluorescence analysis of LINE1 and IAP in testes from adult wild-type (A, D), Mov10l1 fl/- Neurog3-Cre (B, E), and Mov10l1 −/− (C, F) mice. Leydig cells reside in the interstitium and emit a strong autofluorescence signal. In Mov10l1 −/− (ubiquitous knockout) testes, LINE1 (C) and IAP (F) are de-repressed in spermatocytes and spermatogonia, respectively [22], providing a positive control . In contrast, LINE1 (B) and IAP (E) are not de-repressed in Mov10l1 fl/- Neurog3-Cre testes. Scale bar, 50 µm. (TIF) [file pgen.1003038.s006.tif]
