# Supplementary material for: Blockade of Pachytene piRNA Biogenesis Reveals a Novel Requirement for Maintaining Post-Meiotic Germline Genome Integrity
Source: PLoS Genet. 2012 Nov 15;8(11):e1003038. doi: 10.1371/journal.pgen.1003038 (PMC3499362; doi:10.1371/journal.pgen.1003038)
Supplement: Table S1 — Testis weight, sperm production, and fertility of wild type and Mov10l1 fl/- Prm-Cre (mutant) male mice. (DOC) [file pgen.1003038.s008.doc]

**Table S1** **Testis weight, sperm production, and fertility of wild type and *Mov10l1*^fl/-^ Prm-Cre (mutant) male mice^a^**

|  | Genotype | |  |  |
| --- | --- | --- | --- | --- |
|  | WT^b^ | mutant^b^ | Ratio of mutant to WT | P value |
| Body weight (g) | 25.9 ± 2.1 | 29.1 ± 5.5 | 1.12 | 0.32 |
| Testicular weight (mg) | 217.7 ± 14.0 | 188.1 ± 8.0 | 0.86 | 0.01* |
| Sperm/cauda (10^7^) | 1.82 ± 0.34 | 1.67 ± 0.28 | 0.92 | 0.53 |
| Offspring (pups/litter)^c^ | 10.2 ± 2.6 | 9.8 ± 2.0 | 0.96 | 0.85 |

^a^ Mice from the mixed genetic background were used at 2-3 months of age.

^b^ 4 pairs (n=4) of WT and mutant males were used except mating test.

^c^3 pairs (n=3) of males were used for matings.

*Values were statistically significant (Student’s *t*-test).
