# Supplementary material for: Blockade of Pachytene piRNA Biogenesis Reveals a Novel Requirement for Maintaining Post-Meiotic Germline Genome Integrity
Source: PLoS Genet. 2012 Nov 15;8(11):e1003038. doi: 10.1371/journal.pgen.1003038 (PMC3499362; doi:10.1371/journal.pgen.1003038)
Supplement: Table S2 — PCR primers for semi-quantitative RT-PCR and qPCR assays. (DOC) [file pgen.1003038.s009.doc]

| Target | Primer name | Primer sequence | PCR cycles* | Product (bp) |
| --- | --- | --- | --- | --- |
| pre-piR1 | pre-piR1-F1 | GTTAGCGAAGGACATTATTCTAACC | 27 | 501 |
|  | pre-piR1-R1 | TGACATGAACACAGGTGCTCAGAT |  |  |
| pre-piR2 | pre-piR2-F5 | CTATGCTTATGATGGCATTGGAGAG | 27 | 530 |
|  | pre-piR2-R4 | TTCCAGTTCAACAGGGACACGGGAC |  |  |
| pre-piR3 | pre-piR3-F1 | GTTCTCACTTTATCAGCTCTCAAG | 27 | 517 |
|  | pre-piR3-R1 | TGAGAGTGGCATCTAAATGTTTAG |  |  |
| pre-piLR | pre-piRNA2-F | GTGAAGCTAAGGATGCTGGGATAG | 27 | 413 |
|  | pre-piRNA2-R | ACAGGATGTCCCCTGAAATCAGTC |  |  |
| Prepachytene cluster 10 | pre-piRNA13-F | GGCCATAGGTTAACTTTCAGAAGTC | 31 | 374 |
|  | pre-piRNA13-R | CTATAACTGCAAGTTCAGGTGGACAG |  |  |
| Line1 | Line1-F1 | GAGAACATCGGCACAACAATC | 20 | 762 |
|  | Line1-R1 | TTTATTGGCGAGTTGAGACCA |  |  |
| pri-let7g | pri-let-7gF | GTACGGTGTGGACCTCATCA | 27 | 137 |
|  | pri-let-7gR | TCTTGCTGTGTCCAGGAAAG |  |  |
| *Sycp2* | Sycp2-13 | TCACCAATGGGAAGCTGTCA | 27 | 390 |
|  | Sycp2-10 | TTGCCTAGTTCTAGAGGGTTT |  |  |
| *Actb* | Actin-3 | AGAAGAGCTATGAGCTGCCT | 25 | 382 |
|  | Actin-4 | TCATCGTACTCCTGCTTGCT |  |  |

**Table S2 PCR primers for semi-quantitative RT-PCR and qPCR assays**

*Number of cycles used for semi-quantitative RT-PCR analysis in Figure 6b.
